# An Increase in Imported *Plasmodium vivax* Malaria in New York City: Clinical and Demographic Trends Following Recent Migration

**DOI:** 10.1093/ofid/ofag293

**Published:** 2026-05-16

**Authors:** Cesar G Berto, Maria Alyssa Policarpio, Coral Vargas-Pena, Mike Antwi, Sally Slavinski, Asha Abdool, Renee N King, Tsoline Kojaoghlanian, Johanna P Daily, Nishant Prasad, Maria R Velasquez, Alison D Ridpath, Christina M Coyle

**Affiliations:** Department of Internal Medicine, Division of Infectious Diseases, University of Alabama at Birmingham, Birmingham, Alabama, USA; Department of Medicine, NewYork City Health + Hospitals/Jacobi, Albert Einstein College of Medicine, Bronx, New York, USA; Department of Medicine, NewYork City Health + Hospitals/Jacobi, Albert Einstein College of Medicine, Bronx, New York, USA; The Bureau of Communicable Disease, NewYork City Department of Health & Mental Hygiene, New York, New York, USA; The Bureau of Communicable Disease, NewYork City Department of Health & Mental Hygiene, New York, New York, USA; The Bureau of Communicable Disease, NewYork City Department of Health & Mental Hygiene, New York, New York, USA; The Bureau of Communicable Disease, NewYork City Department of Health & Mental Hygiene, New York, New York, USA; Division of Pediatric Infectious Diseases, Maimonides Children's Hospital of Brooklyn, Brooklyn, New York, USA; Division of Infectious Diseases, Montefiore Medical Center/Albert Einstein College of Medicine, Bronx, NewYork, USA; Division of Infectious Diseases, NewYork-Presbyterian Queens Hospital, Flushing, New York, USA; Division of Infectious Diseases, NewYork City Health + Hospitals/Metropolitan, New York, New York, USA; Malaria Branch, Division of Parasitic Diseases and Malaria, National Center for Emerging and Zoonotic Infectious Diseases, US Centers for Disease Control and Prevention, Atlanta, Georgia, USA; Department of Medicine, NewYork City Health + Hospitals/Jacobi, Albert Einstein College of Medicine, Bronx, New York, USA

**Keywords:** imported malaria, *P. vivax*, travel

## Abstract

**Background:**

*Plasmodium vivax* causes over 9 million malaria cases globally. While rare in the United States, New York City (NYC) reports the highest number of travel-associated cases. In 2023, cases peaked alongside the arrival of >265 000 migrants from malaria-endemic regions.

**Methods:**

We retrospectively reviewed *P. vivax* cases reported to the NYC Department of Health (2018–2024) and analyzed clinical data from patients admitted to NYC Health + Hospitals (H + H) over the same period. Cases were confirmed via blood smear, rapid diagnostic test, or polymerase chain reaction (PCR). Data on travel history, presentation, diagnostics, treatment, and outcomes were analyzed.

**Results:**

In NYC, the number of *P. vivax* cases rose from 6 of 206 malaria cases (2.9%) in 2018 to a record 63 of 346 (18.2%) in 2023, with 43 of 250 (17.2%) in 2024. Of 41 H + H admissions, 40 (97.6%) were recent migrants, mainly from South America and Asia. Severe malaria occurred in 10 patients (24.4%), with 5 (12.2%) requiring intensive care. Rapid diagnostic tests were used in 38 cases (93%); PCR had a median 8-day turnaround. Primaquine was prescribed to 32 (78%) after G6PD testing. Relapses occurred in 7 patients (17%), mostly among those not receiving liver-stage therapy.

**Conclusions:**

The rise in *P. vivax* malaria in NYC parallels migration trends from endemic areas. Timely diagnosis and liver-stage therapy are critical to reducing morbidity and relapses. While nearly all cases were imported, delayed species confirmation or incomplete treatment risks potential local transmission, requiring heightened clinician awareness and public health vigilance.

## Teaser

New York City experienced a rise in Plasmodium vivax malaria cases from 2018 to 2024, mirroring migration patterns at that time. Gaps in timely diagnosis and liver-stage therapy led to preventable relapses, highlighting the need for increased clinical vigilance and coordinated public health action to avoid transmission.

Malaria continues to pose a significant burden globally, with an estimated 263 million cases reported worldwide in 2023, according to the WHO [1]. The disease is caused by 5 *Plasmodium* species that infect humans: *P. falciparum*, *P. vivax*, *P. malariae*, *P. ovale*, and *P. knowlesi* [1]. Among these, *P. vivax* accounted for approximately 9.2 million cases in 2023. Notably, *P. vivax* has the widest global distribution, with more than half of the cases reported from the Southeast Asia Region, the Eastern Mediterranean Region, and the Region of the Americas [1–3].

*P. vivax* is characterized by a low level of peripheral parasitemia [4, 5] and the potential to form dormant liver stages, or hypnozoites, which can reactivate months to years later if not treated adequately. This unique feature informs the treatment approach for *P. vivax*, which requires targeting both the active blood stage that causes clinical symptoms and the dormant liver stage [6, 7]. Despite its typically milder clinical course, *P. vivax* causes significant morbidity and can lead to severe malaria and death [4, 8–10].

In the United States, approximately 2000 malaria cases are reported annually; most occur in returning travelers visiting friends and family or immigrants from malaria-endemic countries [11]. While local transmission remains exceedingly rare, the presence of competent *Anopheles* mosquito vectors in several regions maintains a theoretical risk for local transmission. New York City (NYC) reports the highest number of travel-associated malaria cases in the United States, accounting for roughly 80% of all malaria diagnosed in New York State [12]. Because malaria is a reportable condition, the NYC Health Department tracks all cases, the majority of which are treated within NYC Health + Hospital System (H + H), the nation's largest municipal healthcare system. In 2023, NYC reported a record number of malaria cases, characterized by a significant increase in the proportion of *P. vivax* cases. This surge coincides with the influx of 265 500 migrants since 2022 [13], many of whom crossed the Darién Gap in route to the United States [14]. Despite these evolving trends, previous research in NYC has remained limited to macro-level data; a previous study [12] did note the general increase in cases following recent migration but lacked patient-specific clinical data and a focused analysis of *P. vivax*. Consequently, there is a critical need to evaluate these local clinical trends to better inform diagnostic protocols and public health prevention strategies.

This retrospective study reports the temporal trends in the absolute number of *P. vivax* cases and its proportion among total malaria cases in NYC from 2018 to 2024. We also provide a descriptive summary of clinical presentations, malaria diagnostic tests, and treatment outcomes of a subset of *P. vivax* case patients presenting to H + H facilities from 2022 to June 2024.

## METHODS

### Study Design and Setting

#### Population-Based Surveillance

This retrospective study included all *P. vivax* malaria cases reported to the NYC Health Department between 2018 and 2024, limited to cases within the 5 boroughs of NYC. Positive malaria test results are reportable to the NYC Health Department by clinical laboratories using an electronic laboratory reporting system, and each report triggers a public health investigation to collect demographic information, clinical course and risk factors for infection, including travel history, to identify potential locally acquired cases.

Initial diagnosis is established at the clinical laboratory level using peripheral blood smear microscopy and/or rapid diagnostic tests (RDTs), with treatment decisions based on these results. Diagnostic specimens are subsequently forwarded to the New York State Wadsworth Center for confirmatory testing and species identification using blood smear microscopy and polymerase chain reaction (PCR), as well as antimalarial resistance testing. New York City Health Department staff also follow up with patients and providers to ensure appropriate therapy, including treatment of latent stage infections associated with *P. vivax*.

#### Clinic-Based Study

We retrospectively retrieved and analyzed patient-level data for all *P. vivax* malaria cases admitted to NYC H + H facilities, from January 2018 and June 2024, including patients initially seen at nearby non-H + H hospitals who were transferred for care. Patients were identified from the electronic medical record (EMR) using the diagnostic codes ICD-9 (084) for malaria or ICD-10 (B51) for *P. vivax* malaria.

Cases were included only if *P. vivax* infection was confirmed by laboratory testing using one of the following methods: a positive blood smear confirming *P. vivax* species, a positive smear for *Plasmodium* spp. with subsequent species confirmation via PCR testing, or RDT followed by PCR confirmation for *P. vivax* in patients with clinical suspicion of malaria. Patients were excluded if species identification could not be confirmed by smear or PCR, or if testing identified a different *Plasmodium* species without evidence of *P. vivax* coinfection.

Baseline demographic variables included sex and comorbidities. Epidemiologic and travel information was collected, including country of birth, reason for travel, migration path by country, and travel dates. Hospitalization data were also gathered, including length of stay, clinical presentation, highest level of care, and outcome on discharge. Severe malaria was classified according to the Centers for Disease Control and Prevention (CDC) criteria [15]. The diagnostic and treatment variables collected included malaria laboratory testing, G6PD testing, antimalarial treatment regimen for both blood and liver stages, CYP2D6 pharmacogenetic testing, and disease relapse. According to CDC guidelines [16], the standard anti-relapse (liver stage) treatment dose of primaquine was 30 mg daily for 14 days in patients weighing less than 70 kg or a total dose of 6 mg/kg in patients weighing over 70 kg. Relapses were defined as the recurrence of *P. vivax* infection, confirmed by a parasite smear, occurring 21 days or more after completing blood-stage treatment [17].

### Laboratory Diagnosis

Parasitological laboratory tests for malaria were extracted from the EMR, including blood smear microscopy, malaria RDT, and PCR. Tests were ordered according to each local hospital's protocol.

### Statistical Analysis

Descriptive statistics for continuous variables were reported as mean ± standard deviation or median with interquartile range, as appropriate; categorical variables were summarized as percentages. Time to migration measured from departure from the home country to arrival in NYC was reported. Statistical analyses were conducted using Stata 13. Ethical approval for the study was obtained from Biomedical Research Alliance of New York under IRB file #23-12-141-719, with a waiver of informed consent granted given the retrospective design and use of de-identified data.

## RESULTS

### Population-Based Surveillance

**Figure 1.**
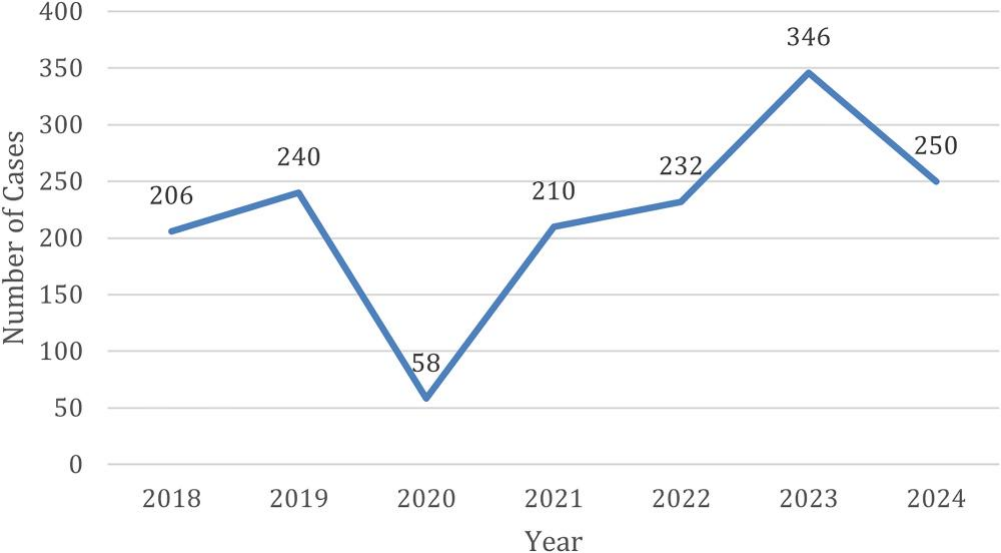
Number of reported total malaria cases in New York City, 2018–2024.

**Figure 2.**
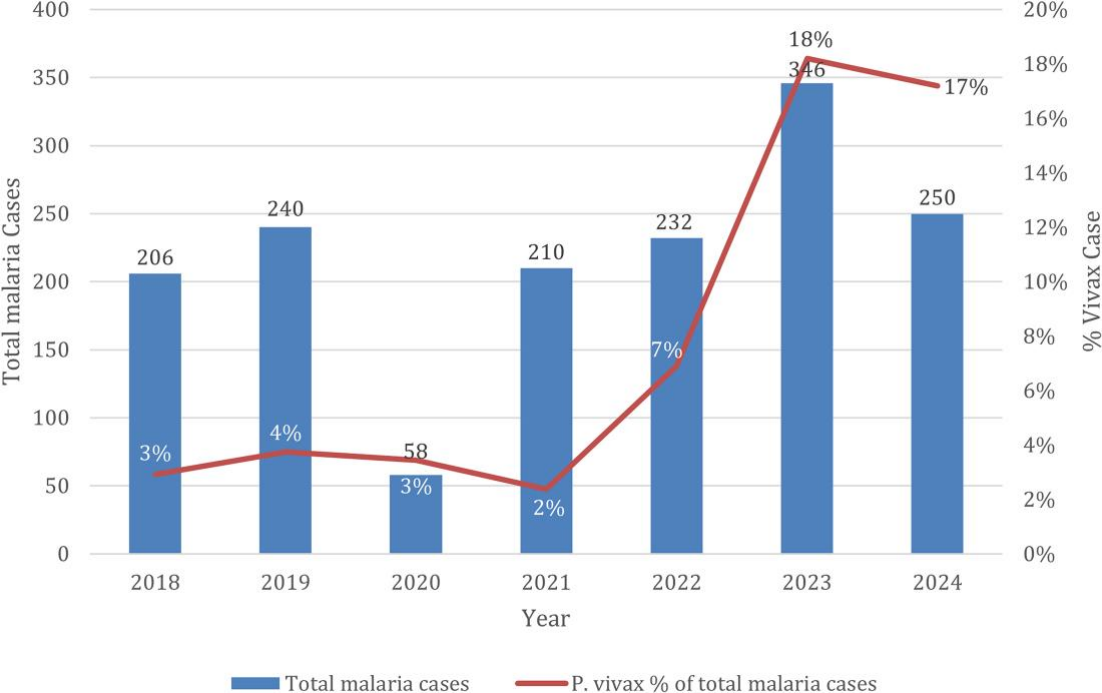
Trend in total malaria cases and proportion attributable to *P. vivax* in New York City, 2018–2024.

The number of malaria cases in NYC increased from 206 in 2018 to 346 in 2023, the highest ever recorded for NYC, followed by a decline to 250 in 2024 (Figure 1). Cases due to *P. vivax* rose in parallel, from 6 of 206 cases in 2018 to a peak of 63 of 346 cases in 2023, before decreasing slightly to 43 of 250 cases in 2024 (Figure 2). Accordingly, the proportion of *P. vivax* among all malaria cases increased from 2.9% in 2018 to 18.2% in 2023, with a modest decline to 17.2% in 2024. The proportion of patients with *P. vivax* who were hospitalized during ranged from 50% to 85.7% over the study period, reaching 85.7% in 2023 and 83.7% in 2024 (Table 1).

**Table 1.** Hospital Admissions for Patients Diagnosed With Malaria Due to *P. vivax*^a^ by Year in New York City Between 2018 and 2024

Admitted	2018	2019	2020	2021	2022	2023	2024	Total
No, n (%)	1 (16.7)	3 (33.3)	1 (50.0)	2 (40)	3 (18.8)	9 (14.3)	7 (16.3)	26 (18.1)
Yes, n (%)	5 (83.3)	6 (66.6)	1 (50.0)	3 (60)	13 (81.3)	54 (85.7)	36 (83.7)	118 (81.9)
Total	6	9	2	5	16	63	43	144

^a^Hospital admission includes those to any hospital, not limited to NYC H + H.

### Clinic-Based Study

The in-depth clinic-based study identified 46 cases of *P. vivax* malaria admitted to H + H over the study period between 2018 and 2024. Among these, 5 cases were diagnosed between 2018 and 2019, and no cases were reported between 2020 and 2021. The remaining 41 cases, diagnosed from 2022 to 2024, are described in detail in Table 2. Most of these patients were male (75.6%), with a mean age of 32.6 ± 10.9 years. Most individuals were born in South America (58.5%), followed by Asia (34.1%) and Africa (7.3%). Only one patient traveled to an endemic area visiting friends and relatives, while the remaining (97.6%) were migrants to the United States. The migration patterns varied among the individuals. Three people (7.3%) traveled by airplane, including 1 visiting friends and relatives who acquired malaria in Pakistan and 2 who flew directly from Chad and Bangladesh. The rest traveled by land; 61% came by land routes only through Colombia, Central America, and Mexico, and 29.3% followed a hybrid migration path, initially flying to South America and then traveling by land through the previously mentioned regions. The median duration between arrival in NYC and the onset of symptoms was 4 days (−36 to +186 days).

**Table 2.** Demographic Characteristics of Patients Diagnosed With Malaria Due to *P. vivax* Evaluated at New York City Health + Hospitals, New York City (NYC) Jan 2022-Jun 2024

Characteristics	N = 41 (%)
Sex, male	31 (75.6)
Age, years (mean ± SD)	32.6 ± 10.9
Region of birth	
South America	24 (58.5)
Asia	14 (34.1)
Africa	3 (7.3)
Travel history	
Reason for travel	
Visiting friends and family	1 (2.4)
Migration	40 (97.6)
Route of travel	
Directly to United States by airplane	3 (7.3)
By land (through Colombia, Central America, and Mexico)	25 (61.0)
Airplane to South America and then by land (through Colombia, Central America, and Mexico)	12 (29.3)
Time from NYC arrival to onset of symptoms, days	4 [−36–186]
Medical encounter preceding confirmed diagnosis	14 (34.1)
Emergency department/admission	10 (24.4)
Urgent care	1 (2.4)
Primary care provider	2 (4.9)
Other	1 (2.4)
Clinical presentation	
Fever/chills	37 (90.2)
Myalgia	27 (65.9)
Headache	21 (51.2)
Nausea/vomiting	25 (61.0)
Abdominal pain	18 (43.9)
Anorexia	12 (29.3)
Diarrhea	9 (22.0)
Met criteria of severity^a^	10 (24.4)
Shock	8 (19.5)
Severe anemia	2 (4.9)
Jaundice	3 (7.3)
Admitted to intensive care unit	5 (12.2)
Diagnostic test	
Microscopy	41 (100)
Time from admission to smear ordered, days	0 [0–4]
Incorrect species reported	3 (7.3)
Negative	3 (7.3)
Reported as *P. vivax* or non-falciparum malaria	35 (85.4)
Malaria rapid diagnostic tests	38 (92.7)
Non-*falciparum* malaria	31 (75.6)
*P. falciparum* malaria	1 (2.4)
Negative	6 (14.6)
PCR	38 (92.7)
Time from PCR ordered to PCR resulted, days	8 [2–22]
Index episode	
Blood stage treatment	
Artemether/lumefantrine	22 (53.7)
Atovaquone/proguanil	10 (24.4)
Chloroquine or hydroxychloroquine	8 (19.5)
Artesunate	1 (2.4)
Quantitative G6PD testing done	40 (97.6)
Normal	39 (95.1)
Deficient	1 (2.4)
Liver stage treatment	32 (78.0)
Primaquine	32 (78.0)
Time from end of blood stage to beginning of liver stage treatment, days	4 [0–59]
Outcome	
Mortality	0
Patients who experienced relapse^b^	7 (17.1)
Treatment	
Chloroquine or Hydroxychloroquine	4 (9.8)
Artemether/lumefantrine	1 (2.4)
Required admission	4 (9.8)
Reason for relapse	
Not treated for the liver stage on the initial episode	5 (12.2)
Subtherapeutic primaquine blood levels	2 (4.9)

Abbreviations: SD, standard deviation; PCR, polymerase chain reaction; G6PD, glucose-6-phosphate dehydrogenase.

^a^Only criteria met by at least one patient are listed. No patients met other WHO severe malaria criteria.

^b^Eight individuals were lost in follow-up.

Fever at initial presentation occurred in 90.2% of patients, followed by myalgias (65.9%) and nausea/vomiting (61%). Of note, 14 patients (34.1%) had a prior medical encounter before admission, during which they presented with similar symptoms. Most patients (61%) were admitted to general medicine floors, with a median length of stay of 4 days (0–13 days). Ten patients (24.4%) met the criteria for severe disease at admission, and 5 of these patients were subsequently admitted to the intensive care unit (ICU). Among those admitted to the ICU, 4 required vasopressor support, and 1 patient received intravenous artesunate. Among the 5 patients managed on the general medicine wards, 2 had severe anemia and 3 had hypotension responsive to fluid therapy.

Microscopy of blood smears was performed in all 41 cases. Among these, 35 (85.4%) were reported as *P. vivax*, 3 (7.3%) were reported erroneously as other non-*Plasmodium vivax* species, and 3 (7.3%) were initially reported as negative. Those with a negative blood smear had a positive rapid malaria diagnostic test, and subsequently, *P. vivax* was confirmed by PCR testing. Thirty-eight patients (92.7%) had a rapid malaria diagnostic test performed in addition to a blood smear. Of them, 31 (75.6%) were reported as non-*Plasmodium falciparum* species, 6 (14.6%) were negative, and 1 (2.4%) resulted as positive for *P. falciparum*. Finally, 38 patients (92.7%) were PCR positive for *P. vivax* with a median time to result of 8 days (2–22 days).

Different antimalarial drugs were used as blood-stage treatment. Chloroquine or hydroxychloroquine, artemether-lumefantrine, and atovaquone-proguanil were administered to 19.5%, 53.7%, and 24.4% of patients, respectively. One patient with severe malaria received intravenous artesunate for treatment, followed by oral antimalarial therapy. G6PD testing was done in 40 patients, and 39 of them had a normal G6PD function. The median turnaround time for G6PD testing was 4 days (0–9 days). Anti-relapse treatment with primaquine was given to 32 of 41 patients (78.0%), with a median interval between blood-stage and liver-stage treatments of 4 days (0–59 days). Among those receiving primaquine, the blood-stage regimens used were artemether/lumefantrine (n = 19), atovaquone/proguanil (n = 8), and chloroquine or hydroxychloroquine (n = 5).

Relapses occurred in 7 patients (17.1%) with a median of 52 days (IQR 25–70) after completing blood-stage treatment; 4 of these required hospital readmission. Five patients did not receive liver-stage treatment and were subsequently retreated and given primaquine for liver-stage therapy. One patient had received a subtherapeutic dose of liver-stage treatment, and another was found to have a CYP2D6 heterozygous allele (see Table 3). These 2 patients required retreatment with an extended course of primaquine, dosed at a total of 6 mg/kg. The duration of daily 30 mg dosing was extended to complete the calculated total dose.

**Table 3.** Clinical Characteristics of the Patients Who Experience *P. vivax* Relapse Infection NYC HHS, NYC Jan 2022–Jun 2024

	Case 1	Case 2	Case 3	Case 4	Case 5	Case 6	Case 7
Initial blood stage treatment Duration	Artemether-lumefantrine 3 d	Atovaquone-proguanil 4 d	Artemether-lumefantrine 3 d	Hydroxychloroquine	Artemether-lumefantrine 3 d	Artemether-lumefantrine 3 d	Atovaquone-proguanil 4 d
Initial liver stage treatment	Primaquine 30 mg once daily for 14 d, initiated 1 d prior to completing blood-stage therapy	No	No	No	Primaquine 30 mg once daily for 17 d, initiated 4 d after completing blood-stage therapy	No	No
Reason for no liver stage treatment during the first episode	N/A	Lost to follow-up after discharge	Lost to follow-up after discharge	Lost to follow-up after discharge	N/A	Delayed initiation of liver stage	Lost to follow-up after discharge
Number of days between relapse and blood stage treatment	70 d	52 d	21 d	26 d	62 d	25 d	86 d
Treatment for relapse	Hydroxychloroquine 800 mg, then 400 mg × 3 d Primaquine 52.6 mg (30 mg base) × 20 d	None Primaquine 52.6 mg (30 mg base) × 14 d	Chloroquine 600 mg then 300 mg × 3 doses Primaquine 52.6 mg (30 mg base) × 14 d	Hydroxychloroquine 800 mg, then 400 mg × 3 doses Primaquine 52.6 mg (30 mg base) × 14 d	Artemether-lumefantrine × 2 d Hydroxychloroquine 800 mg, then 400 mg × 3 doses Primaquine 52.6 mg (30 mg base) × 17 d	Chloroquine 500 mg × 5 d Primaquine 52.6 mg (30 mg base) × 14 d	Hydroxychloroquine 800 mg × 1, atovaquone-proguanil × 3 d Primaquine 52.6 mg (30 mg base) × 14 d
CYP2D6 tested	Yes Normal	No	No	No	Yes Heterozygous for 2D6*10 dec func allele	No	No
Suspected reason for relapse	Initial primaquine dose was likely insufficient due to the patient's weight (>80 kg). For relapse, the treatment was extended to 20 d at 30 mg daily	Liver-stage treatment was not given after the first episode Primaquine was prescribed after the second episode	Liver-stage treatment was not given after the first episode Primaquine was prescribed after the second episode	Liver-stage treatment was not given after the first episode Primaquine was prescribed after the second episode	CYP2D6 genotyping revealed that the patient was an intermediate metabolizer, which may have impaired primaquine activation and contributed to relapse despite extended weight-based treatment. He was well at a follow-up 10 d after finishing the second 17-d primaquine course	Liver-stage treatment was not given after the first episode Primaquine was prescribed after the second episode	Liver-stage treatment was not given after the first episode Primaquine was prescribed after the second episode

## DISCUSSION

This retrospective review of *P. vivax* cases in NYC revealed a large increase in the number of cases in 2023 compared to prior years, aligning with the rising influx of migrants traveling through South and Central America into the United States [18]. Likewise, in 2023, an increase in *P. vivax* cases was reported in the southern border states [19] and healthcare systems serving newly arrived immigrants in California [20]. Similar trends were also observed in Europe between 2010 and 2016, where a significant rise in malaria cases coincided with the influx of Eritrean migrants [21, 22].

The time between arrival and symptom onset varied widely, with some patients developing symptoms months after arrival, while others had prolonged symptoms before receiving a diagnosis. The longer incubation periods observed in our series align with prior reports, which found that up to 40% of *P. vivax* cases had symptom onset 90 or more days after returning to the United States [11]. Despite prior healthcare visits, up to 40% of patients experienced a delay in diagnosis, possibly due to the nonspecific nature of their symptoms. As seen in other reports, gastrointestinal symptoms were common [23] and may have hindered early identification. Paucisymptomatic infections, coupled with extended intervals from migration to symptom onset, could contribute to local malaria transmission, adding a critical layer of complexity to public health efforts. These findings highlight the importance of early detection of malaria among recent arrivals and underscore the need for heightened awareness and proactive screening in immigrant populations [11, 24].

Over the past few decades, there has been increasing evidence that *P. vivax* can lead to severe illness and mortality [3, 4, 7, 25]. In our study cohort, 10 patients (24.4%) met CDC criteria for severe malaria, and 5 required ICU admission. Despite this, no deaths occurred. In comparison, the CDC reported that in 2018, only 4.6% of *P. vivax* cases were classified as severe [11]. The difference in the proportion of severe cases may be attributed to delays in diagnosis and treatment, which are associated with worsened outcomes and more severe manifestations [26]. Healthcare provider's inability to diagnose malaria contributes significantly to this delay [27]. Furthermore, migrants' fear of accessing healthcare, along with malnutrition and comorbidities, may delay seeking medical attention [28, 29] and increase the risk for severe illness. Targeted outreach to educate migrants about malaria and the importance of prompt testing and treatment, coupled with healthcare provider training to enhance early suspicion, are crucial to closing this gap.

Direct visualization of blood smears requires highly trained laboratory technicians, which can lead to inaccurate species identification [30]. In our cohort, up to 25% of cases could not be identified to the species level with microscopy alone; 3 cases were misidentified as a different species, and 1 blood smear was initially reported as negative. This aligns with previous reports highlighting the limited sensitivity of blood smears, where misclassification of malaria species is particularly common in non-*falciparum* infections [31]. While the sensitivity of RDTs is excellent for *P. falciparum*, previous studies [32], consistent with our findings, have reported a lower sensitivity for non-*falciparum* species. Additionally, most RDTs cannot differentiate between non-*falciparum* species or detect mixed infections. These limitations in species identification may have important treatment implications, as misclassification could result in unnecessarily broad antimalarial therapy or failure to provide appropriate liver-stage treatment. In such cases, PCR testing was essential for confirming species diagnosis and informing of the need for liver-stage treatment. Notably, PCR was performed in 91% of patients in this cohort, a scenario that may not reflect the test's availability in other regions of the United States.

As timely species-level diagnosis was not available for most patients, most patients received an artemisinin-based combination therapy, likely chosen due to its efficacy against all malaria species. Intravenous artesunate is the treatment for severe malaria [33, 34], and 1 patient among the 10 who met the criteria for severe malaria received it, suggesting either a lack of recognition of optimal treatment or the perception of *P. vivax* as less severe compared to *P. falciparum*. G6PD status should be assessed before administering primaquine [7], but delays in test results can impact timely treatment. In our cohort, the median turnaround time was 4 days, and 22% of patients did not receive primaquine, partly due to loss of follow-up after receiving their results. Among them, 5 patients experienced a relapse. Of those who received primaquine, 2 relapsed: 1 due to underdosing and 1 due to a CYP2D6 heterozygous allele. Current recommendations emphasize the importance of administering treatment based on the total dose of primaquine given throughout the entire treatment course, rather than using a standard duration of 14 days, to optimize treatment effectiveness [35].

While this study provides valuable insights, several limitations must be acknowledged. Its retrospective design restricts systematic data collection to information only from clinical encounters within NYC H + H system and relies solely on available documentation for cases requiring admission. This could also have resulted in an underreporting of relapses, as patients might have been briefly in NYC, chosen not to seek medical care, or sought treatment at another hospital. Additionally, as our cases were obtained from patients presenting to the major safety net hospitals in NYC, there is a potential for selection bias toward a population that is more severely ill, symptomatic, and underinsured.

Our findings indicate that the increase in *P. vivax* cases in NYC corresponded to the increased migration of individuals from and/or through malaria-endemic regions. The clinical presentation of malaria is variable, and clinicians must have a high index of suspicion when taking care of these populations. Although malaria diagnostics have become more accessible, significant delays in obtaining results can critically impact treatment decisions. To effectively address these challenges, healthcare agencies must adopt a multifaceted strategy that includes raising awareness about malaria among healthcare professionals and migrant communities, speeding up diagnostic turnaround times, and implementing robust treatment protocols.
